# SARS-CoV-2 Infection, Vaccination and Risk of Death in People with An Oncological Disease in Northeast Italy

**DOI:** 10.3390/jpm13091333

**Published:** 2023-08-29

**Authors:** Lucia Mangone, Paolo Giorgi Rossi, Martina Taborelli, Federica Toffolutti, Pamela Mancuso, Luigino Dal Maso, Michele Gobbato, Elena Clagnan, Stefania Del Zotto, Marta Ottone, Isabella Bisceglia, Antonino Neri, Diego Serraino

**Affiliations:** 1 Epidemiology Unit, AUSL-IRCCS di Reggio Emilia, 42122 Reggio Emilia, Italy; lucia.mangone@ausl.re.it (L.M.); pamela.mancuso@ausl.re.it (P.M.); isabella.bisceglia@ausl.re.it (I.B.); 2 Unit of Cancer Epidemiology, Centro di Riferimento Oncologico di Aviano (CRO) IRCCS, 33081 Aviano, Italy; mtaborelli@cro.it (M.T.); ftoffolutti@cro.it (F.T.); dalmaso@cro.it (L.D.M.); serrainod@cro.it (D.S.); 3 Agenzia Regionale di Coordinamento per la Salute Udine, 33100 Udine, Italy; michele.gobbato@arcs.sanita.fvg.it (M.G.); elena.clagnan@arcs.sanita.fvg.it (E.C.); stefania.delzotto@arcs.sanita.fvg.it (S.D.Z.); 4 Scientific Directorate, AUSL-IRCCS di Reggio Emilia, 42122 Reggio Emilia, Italy; antonino.neri@ausl.re.it

**Keywords:** COVID-19, vaccination, risk of death, tumors

## Abstract

People with a history of cancer have a higher risk of death when infected with SARS-CoV-2. COVID-19 vaccines in cancer patients proved safe and effective, even if efficacy may be lower than in the general population. In this population-based study, we compare the risk of dying of cancer patients diagnosed with COVID-19 in 2021, vaccinated or non-vaccinated against SARS-CoV-2 and residing in Friuli Venezia Giulia or in the province of Reggio Emilia. An amount of 800 deaths occurred among 6583 patients; the risk of death was more than three times higher among unvaccinated compared to vaccinated ones [HR 3.4; 95% CI 2.9–4.1]. The excess risk of death was stronger in those aged 70–79 years [HR 4.6; 95% CI 3.2–6.8], in patients with diagnosis made <1 year [HR 8.5; 95% CI 7.3–10.5] and in all cancer sites, including hematological malignancies. The study results indicate that vaccination against SARS-CoV-2 infection is a necessary tool to be included in the complex of oncological therapies aimed at reducing the risk of death.

## 1. Introduction

Since the beginning of the COVID-19 pandemic, it has been documented that people with an oncological disease, both those in active treatment and those undergoing periodic follow-up checks, are at an elevated risk of severe COVID-19 and further sequelae [1,2,3,4]. For these people, biological (related to the disease and/or anticancer treatments) and organizational factors combine to expose them to significantly higher risks of hospital admissions for COVID-19 and death as compared to corresponding people without cancer [5,6,7].

The relative effect of cancer on COVID-19 prognosis is stronger in younger patients [6]. Giannakoulis and colleagues confirmed that COVID-19 cancer patients are at increased risk of dying than non-cancer patients [HR 1.66; 95% CI 1.3–2.1] and that such elevated risk tends to reduce at over 65 years of age [HR 1.06; 95% CI 0.8–1.4] [8], as for other comorbidities [9]. Furthermore, haematological tumors can represent a negative prognostic factor compared to solid tumors, especially in response to therapies [10], but also for a complete immunization after vaccination [11].

On the contrary, evidence about the association between cancer and infection is less conclusive [6,12]. Consistent with this evidence of elevated health risks, people with a history of oncological disease have been included in the high-priority population groups for vaccination against SARS-CoV-2 infection [13,14]. Several studies, including national ones, have already documented the impact of SARS-CoV-2 infection on the mortality of people with cancer [6,15,16].

Most of the studies studied the efficacy of vaccination in cancer patients recruited in oncological services who do not represent the total population living after a cancer diagnosis. It is important to assess the impact of vaccination in the population of cancer patients across all the phases of the disease and cure and across all cancer sites in large population-based studies. A vaccination campaign in Italy started on December 2020, targeting initial health operators and residents in nursing homes. Older and vulnerable people were vaccinated with a high priority [17] and reached a high two-dose coverage by the end of March 2021. People with a previous diagnosis of cancer were included in the vulnerable group and were mostly vaccinated with an mRNA vaccine.

The aim of the study is to assess the risk of death in cancer patients based on their SARS-CoV-2 infection history and vaccination status.

## 2. Materials and Methods

### 2.1. Setting and Study Design

A retrospective population study was conducted in all residents of the Friuli Venezia Giulia Region and the province of Reggio Emilia living on 1 January 2021. They were therefore eligible for anti-SARs-CoV-2 vaccination and targeted to a molecular swab search for SARS-CoV-2 infection between 1 January 2021 and 31 December 2021. This study compared the risk of death for any cause in patients who tested positive for SARS-CoV-2 in 2021 at least once.

### 2.2. Data Sources and Linkage Procedures

**Figure 1:**
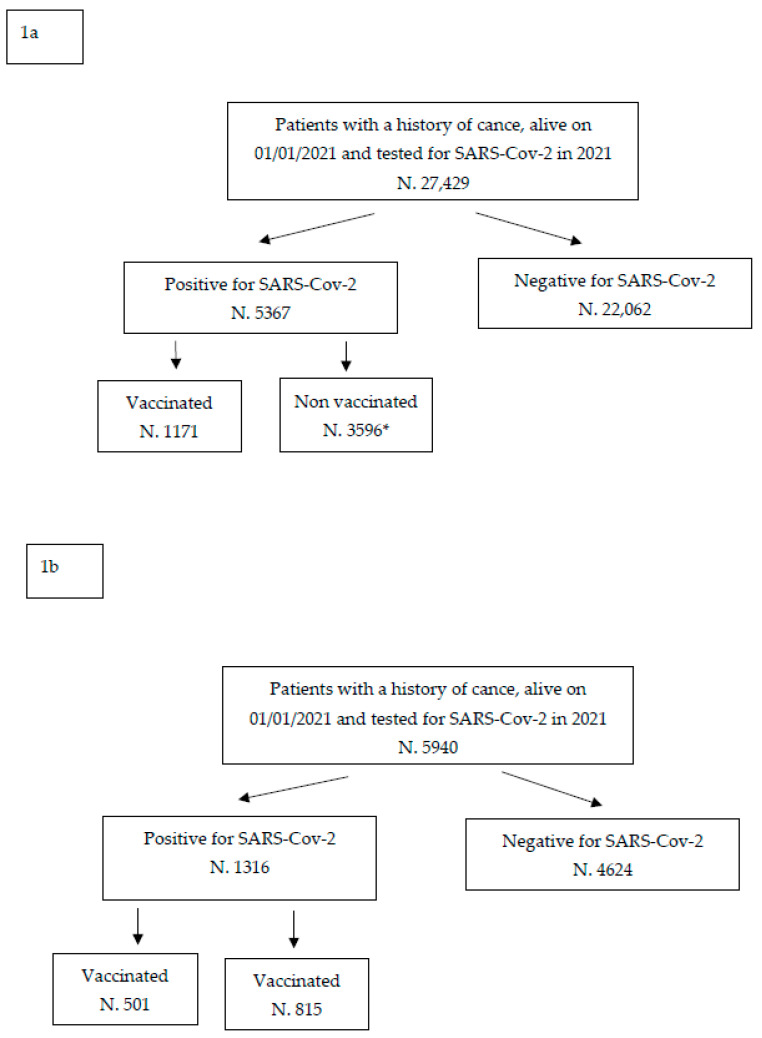
Description of the study population: Friuli Venezia Giulia (**1a**) and Reggio Emilia (**1b**) * It includes 2273 patients who received their first dose after SARS-CoV-2 positive test.

To this end, the anonymised data included in the databases of the regional health information system were used for the Friuli Venezia Giulia Region, which covers the entire resident population [18]. For the province of Reggio Emilia, anonymised data from the Population Cancer Registry (CR) were used [19]. For the Friuli Venezia Giulia region, the initial population consisted of 725,475 residents who underwent a nasopharyngeal swab for the detection of SARS-CoV-2, of which 27,429 who tested positive had a previous history of cancer. For Reggio Emilia province, the corresponding population with cancer was made up of 5940 individuals (Figure 1a,b).

Persons with all negative swabs were considered SARS-CoV-2 negative on the date of the first swab while those with at least one positive result were considered SARS-CoV-2 positive on the date of the first positive swab. The history of the oncological disease was reconstructed thanks to data from the Reggio Emilia-CR and the Friuli Venezia Giulia Regional CR. For patients with multiple tumours, the most recent diagnosis, before 1 January 2021, was considered. The living status information was updated on 8 January 2022 for Friuli Venezia Giulia or on 30 March 2022 for Reggio Emilia. The study, approved by the Bioethics Committee of the Veneto Region (protocol No. 245343/2020) and by the Area Vasta Emilia Nord Ethic Committee (protocol No. 2020/0045199), was conducted through a record linkage procedure of de-identified data with the use of a semi-annually modified anonymous individual key. For this analysis, the databases of microbiology laboratories, cancer registries and mortality were used. Study subjects were categorized as vaccinated if they received at least one dose of available vaccine or unvaccinated.

### 2.3. Data Analyses

The risk of death was assessed for unvaccinated versus vaccinated cancer patients, overall and by strata of sex, age, cancer type and time since cancer diagnosis. For the calculation of the risk of death, a multivariate analysis based on the Cox model was conducted aimed at estimating the hazard ratios (HR) and their 95% confidence intervals (CI). Models were adjusted for gender, age and time since diagnosis. The time at risk of death was calculated from the date of SARS-CoV-2 infection to the date of death or the study closure date (i.e., 31 March 2022), vaccination status was considered at the time of SARS-CoV-2 infection, as previously described [20]. To better evaluate the impact according to single tumor site, we also reported the relative survivals (adjusted by causes of death) of patients with cancer diagnoses in the years 2015–2017 and follow-up on 31 December 2021.

A descriptive analysis of the causes of death among vaccinated and unvaccinated patients and of the delay time between infection and death was possible only for the incident cases in the province of Reggio Emilia. Data are listed in the Supplementary Materials.

## 3. Results

An oncological history was documented in 27,429 people residing in Friuli Venezia Giulia living on 1 January 2021 and tested for SARS-CoV-2 infection during 2021. Among these oncological patients, 5367 (19.6%) tested positive at least once for SARS-CoV-2 infection during 2021 while 22,062 always tested negative (80.4%). The subsequent statistical analyses concerned the 5367 positive patients: 1171 vaccinated (21.8%) and 3596 (78.2%) unvaccinated at the time of infection (Figure 1a).

Figure 1b shows similar data documented in the province of Reggio Emilia: among 5940 patients with a previous history of cancer and tested in 2021, 1316 (22.2%) were positive and 4624 (77.8%) were negative for SARS-CoV-2 infection. Among the 1316 positives, 501 (38.1%) received at least one dose of the vaccine, and 815 (61.9%) were unvaccinated.

### 3.1. Frequency of Deaths among Vaccinated and Unvaccinated

In Friuli Venezia Giulia (Table 1), among those positive for the infection and vaccinated before infection, 102 died (5.8%); among those unvaccinated, there were 595 deaths (16.6%). Twenty-four deaths (4.1%) occurred among Reggio Emilia patients (Table 1) who received at least one dose of vaccine before infection. Conversely, there were 79 deaths (9.7%) among unvaccinated patients (Table 1).

As regards the study conducted in the Friuli Venezia Giulia region, particularly an elevated percentage of deaths among vaccinated people were documented in males (7.5%), in those aged 80 years or more (13.6%), in patients diagnosed with cancer in the previous 12 months (8.3%) and in patients with haematological malignancies (8.3%), bladder (10.4%) or colorectal (8.9%) among solid tumors.

In the province of Reggio Emilia (Table 1), high proportions of deaths among vaccinated patients occurred in females (5.7%), in those aged 80 or more years (16.1%) and in patients with hematological malignancies (8.2%), kidney (18.2%) or bladder cancer (8.8%), whereas no trend was observed by the time since diagnosis.

Among the unvaccinated patients in Friuli Venezia Giulia, males showed elevated death risk (20.8%); the death risk increases with age (18.4% in 70–79; 37.5% in 80+) and mainly affected patients diagnosed less than 12 months (28.8%). In addition to haematological tumours (18.7%), the lungs (37.2%) and bladder (26.9%) were among the tumour sites with elevated death percentages. Similarly, in the province of Reggio Emilia, unvaccinated males presented an elevated death risk (11.7%); the risk increased with age, especially to 80+ (35.9%), and mainly affected patients with a diagnosis made less than 12 months (21.3%) prior. Among tumor sites, a greater risk was observed for lung cancer (26.5%) and endometrium (29.6%).

### 3.2. Multivariate Analysis of Deaths among Unvaccinated vs. Vaccinated Patients

The risks of death in unvaccinated vs. vaccinated patients are illustrated in Table 2. In Friuli Venezia Giulia, the risk of death among the unvaccinated was almost three-fold that of the vaccinated ones [HR 2.7; 95% CI 2.2–3.4]. The excess of risk was similar in males and females [HR 2.7; 95% CI 2.1–3.6; and HR 2.7; 95% CI 1.8–3.9] in the age group 70–79 [HR 3.7; 95% CI 2.3–5.9] and in subjects diagnosed between one–two years before diagnosis [HR 4.5; 95% CI 1.8–15.3]. Among solid tumours, the excess risk was particularly marked for lung [HR 5.0; 95% CI 1.8–14.1], breast [HR 3.0; 95% CI 1.6–5.3], prostate [HR 2.8; 95% CI 1.7–4.6] and colorectal [HR 2.3; 95% CI 1.4–3.7] cancers.

For hematologic malignancies, the excess risk was significant [HR 2.4; 95% CI 1.3–4.4].

In the province of Reggio Emilia (Table 2), the risk of death among unvaccinated was eight-fold higher than in vaccinated patients [HR 8.2; 95% CI 5.1–13.2]. The excess risk was higher in males [HR 12.1; 95% CI 6.0–24.5], in the 70–79 age group [HR 19.3; 95% CI 5.7–65] and in subjects diagnosed one year before vaccination [HR 20.7; 95% CI 4.0–10.7]. The excess risk in non-vaccinated people was appreciable for bladder [HR 20.6; 95% CI 1.5–27.5], colorectal [HR 18.2; 95% CI 3.5–95.5], prostate [HR 8.2; 95% CI 2.0–34.1] and breast tumours [HR 7.5; 95% CI 2.1–26.6]. For haematological malignancies, sparse data made it difficult to calculate the HRs.

When pooling data from the two cohorts, the general picture of a large excess risk of death in unvaccinated was confirmed with more than three-fold risks. Differences between sexes almost disappeared, while the higher excess risk in older patients was confirmed, while a clearer trend according to time since cancer diagnosis emerged with a stronger excess risk in newly diagnosed. The excess risk was appreciable for almost all cancer sites, but for skin melanoma, kidney cancer and bladder cancer, the excess was compatible with random fluctuations.

Table 3 shows the survivals one year and five years after a diagnosis of the cases recorded in the years 2015–2017. One year after diagnosis, Friuli Venezia Giulia presents an extremely low survival for lung cancer (44%) and low for leukemia (66%) and colorectal cancer and non-Hodgkin’s lymphoma (82%); at five years, survival drops drastically for the sites studied, except breast, prostate, melanoma and thyroid cancer.

Reggio Emilia confirms an extremely low survival for lung cancer (41%) and low for leukemia (76%) and colorectal cancer and kidney cancer (84%) one year after diagnosis; at five years, survival drops drastically for the sites studied, except for breast, prostate, melanoma and thyroid cancer.

Regarding the causes of death, available only for the province of Reggio Emilia (Supplementary Table S1), of the 103 deaths, 17%, 50% and 33% e 20%, 57% e 23% died from a tumor, COVID-19 or other causes among vaccinated and non-vaccinated patients, respectively. Supplementary Figure S2 instead shows the distribution over time between infection and death. Among the vaccinated, 71% and 88% die within 30 and 60 days of infection, respectively, while among the unvaccinated, the percents are 62% and 70%, respectively.

## 4. Discussion

The results of this longitudinal investigation from two population-based cohorts agree in indicating that unvaccinated cancer patients infected with SARS-CoV-2 have a risk of death approximately three times higher than the corresponding vaccinated cancer patients. When the vaccine was not yet available, the national and international literature demonstrated a higher COVID-19 mortality among patients with cancer compared to the general population [1,2,3,4,5,6,7,15,16].

Specifically, national data [16] showed that cancer patients had a higher chance of being hospitalized (56.6% vs. 34.4%) and dying (14.7% vs. 4.5%) from COVID-19 than the general population, confirmed by a subsequent study [6], which showed a higher risk of hospitalization [OR = 1.27; 95% CI 1.09–1.48] and death [OR = 1.45; 95% CI 1.12–1.89] in cancer patients compared to the general population, especially in the presence of metastases and in tumors diagnosed in the two years preceding the infection. A previous Friuli Venezia Giulia study [15] also confirmed the risk of death [OR = 1.63; 95% CI 1.49–1.78], but not an increased risk of hospitalization (in this case the data refers only to admissions to intensive care). Since their development and dissemination, lower immunogenicity of SARS-CoV-2 vaccines has been demonstrated in patients suffering from various forms of cancer compared to healthy populations, particularly in patients with haematological malignancies and in patients undergoing active treatments [21]. The safety profile was similar in cancer patients and the general population [22]. Vaccines and particularly mRNA vaccines [23] was shown to be effective in protecting cancer patients from severe COVID-19 [22]. It is also well-known that SARS-Cov-2 mRNA vaccines are more effective in the general population than in the tumor population and that among the latter, the immune response in hematological patients is significantly lower than in patients with solid tumors [24].

Following the spread of SARS-CoV-2 vaccines, some studies have analyzed their impact on clinical complications, including death [3,23]. In Europe, the results of the multicenter retrospective study “OnCovid Registry Study” showed a statistically significant 74% reduction in the risk of death 28 days after vaccination for vaccinated cancer patients [5]. Our findings indicate that those vaccinated within two years of a cancer diagnosis (and likely to receiving anti-cancer therapies) were at a higher risk of death—an observation in line with previous reports (1, 5).

In general, our study confirms an excess of death for unvaccinated people compared to vaccinated people, which increases with age and which presents a gradient inversely proportional to the time from cancer diagnosis.

Some of the results that emerged from our study deserve particular attention. To avoid overestimating the impact of the vaccine due to differences in testing and biases in the probability of reporting a diagnosis of COVID-19, we compared the risk of death in vaccinated and unvaccinated cancer patients who had at least one positive test for SARS-CoV-2. Nevertheless, with this approach, we only measure the effect of the vaccine on reducing the severity of the disease once the infection occurred and not the protection due to reducing the probability of having a detectable infection, which was probably also not negligible during 2021 when the dominant viral variants were alpha and delta [23], for which sustained efficacy at least in the 6 to 12 months after vaccination has been demonstrated [23,25,26,27]. In both Friuli Venezia Giulia and Reggio Emilia provinces, the excess risk in unvaccinated cancer patients was larger in elderly people aged 70 or more. Considering that the absolute risk of death and particularly of COVID-19-related death is particularly high in this group, the impact of the vaccine in terms of avoided death is more important than what could be inferred by the average protection of a three-fold reduction observed overall. As well, it is worth noting that the excess risk is highest in patients in one to two years from diagnosis, and it is also well appreciable in the first year after cancer diagnosis, the periods when cancer-related mortality is higher [28]. The stronger protection observed in the most fragile patients is probably due to the early vaccination of these groups, i.e., in January and February 2021, when the alpha peak was rising in Italy and both risks of infection, mortality and fatality rate among reported cases were particularly high [29]. A similar effect has been observed in the general population in Reggio Emilia [19]. Different timing of vaccination relative to different epidemiology of epidemic waves could explain the reason for the different protection observed in Friuli Venezia Giulia and Reggio Emilia. In fact, in Reggio Emilia the proportion of patients who were vaccinated after the infection was higher than in Friuli Venezia Giulia, suggesting that infections in early 2021 were more important than those during autumn 2021 in Reggio Emilia compared to Friuli Venezia Giulia. We know that the vaccine was more effective against alpha infections and that it partially lost its effectiveness in late autumn 2021 [19,25,26,27]. Nevertheless, the protection is high in both cohorts.

As far as individual tumor sites are concerned, an excess risk of death for hematological tumors compared to solid tumors is confirmed in both settings studied. As regards solid neoplasms, Friuli Venezia Giulia shows an excess of deaths among the unvaccinated for lung cancers and, subsequently, breast, prostate and colorectal cancers. For Reggio Emilia, an excess of risk for bladder, colorectal and breast is confirmed. The excess risk for lung cancer, already described in the literature [30], was evident only in Friuli Venezia Giulia, suggesting that early hospitalization in Reggio perhaps had a protective effect on mortality [6].

The effect is appreciable in almost all cancer sites, and the few exceptions are largely compatible with random fluctuations. Our data do not confirm a lower vaccine efficacy in patients with haematological patients [21]. Nevertheless, due to very few deaths, we could not assess differences for specific hematological malignancies.

Among the limitations of the study, we must point out that we have no information on the stage of the tumors or on the severity of the COVID-19 infection. Furthermore, information on comorbidities that may have played an important role in the evolution of the disease is lacking.

Among the strengths of this study is the complete observation of the resident population both in the Friuli Venezia Giulia Region and in the province of Reggio Emilia thanks to the availability of complete and accurate health databases. This allowed for all RT-PCR tests for SARS-CoV-2 to be included during the study period. Another strength of the study was the use of data from two population-based cancer registries with a long history and a high-quality standard in terms of completeness and accuracy of the data collected.

## 5. Conclusions

In conclusion, this investigation shows that vaccination significantly reduces the risk of death of people with cancer who are infected with SARS-CoV-2.

## Figures and Tables

**Table 1:** Numbers of cancer patients and deaths (all causes) in patients who tested positive for SARS-CoV-2 at least once according to vaccine status in 2021 in Friuli Venezia Giulia and Reggio Emilia areas, Italy.

	Positive for SARS-CoV-2							
	Friuli Venezia Giulia				Reggio Emilia			
	Vaccinated		Not Vaccinated		Vaccinated		Not Vaccinated	
	Total	Deaths	Total	Deaths	Total	Deaths	Total	Deaths
	N	N (Row %)	N	N (Row %)	N	N (Row %)	N	N (Row %)
All	1771	102 (5.8)	3596	595 (16.6)	501	24 (4.8)	815	79 (9.7)
Sex								
Male	858	64 (7.5)	1665	346 (20.8)	237	9 (3.8)	341	40 (11.7)
Female	913	38 (4.2)	1931	249 (12.9)	264	15 (5.7)	474	39 (8.2)
Age at infection (years)								
<40	56	0 (0.0)	145	1 (0.7)	43	0 (0.0)	67	0 (0.0)
40–59	364	3 (0.8)	869	25 (2.9)	124	1 (0.8)	289	6 (2.1)
60–69	285	6 (2.1)	710	61 (8.6)	88	2 (2.3)	149	4 (2.7)
70–79	522	19 (3.6)	1017	187 (18.4)	134	3 (2.2)	178	22 (12.4)
≥80	544	74 (13.6)	855	321 (37.5)	112	18 (16.1)	131	47 (35.9)
Time since cancer diagnosis								
<1 year	168	14 (8.3)	354	102 (28.8)	40	2 (5.0)	75	16 (21.3)
1–2 years	128	5 (3.9)	298	59 (19.8)	41	0 (0.0)	87	8 (9.2)
2–5 years	365	21 (5.8)	735	95 (12.9)	136	7 (5.2)	221	9 (4.1)
>5 years	1110	62 (5.6)	2209	339 (15.4)	284	15 (5.3)	432	46 (10.6)
Tumor site								
Solid tumors	1614	89 (5.5)	3275	535 (16.3)	440	19 (4.3)	847	130 (15.3)
Breast	415	13 (3.1)	882	84 (9.5)	118	4 (3.4)	222	14 (6.3)
Prostate	284	20 (7.0)	539	109 (20.2)	54	3 (5.6)	77	9 (11.7)
Colorectal	236	21 (8.9)	434	85 (19.6)	53	2 (3.8)	67	10 (14.9)
Skin Melanoma	148	6 (4.1)	287	18 (6.3)	42	2 (4.8)	53	2 (3.8)
Lung and larynx	73	4 (5.5)	180	67 (37.2)	12	0 (0.0)	34	9 (26.5)
Thyroid	91	1 (1.1)	155	10 (6.5)	43	0 (0.0)	74	1 (1.4)
Kidney	63	3 (4.8)	141	19 (13.5)	11	2 (18.2)	29	1 (3.4)
Bladder	48	5 (10.4)	93	25 (26.9)	34	3 (8.8)	44	5 (11.4)
Endometrium	47	1 (2.1)	107	13 (12.2)	15	0 (0.0)	27	8 (29.6)
Other solid tumours	209	15 (7.2)	457	105 (23.0)	58	3 (5.2)	109	19 (17.4)
Hematological malignancies	157	13 (8.3)	321	60 (18.7)	61	5 (8.2)	79	1 (1.3)
Non-Hodgkin lymphoma	73	5 (6.9)	165	38 (23.0)	24	2 (8.3)	38	1 (2.6)
Leukaemia	42	4 (9.5)	65	12 (18.5)	20	2 (10.0)	28	0 (0.0)
Hodgkin’s lymphoma	21	2 (9.5)	51	2 (3.9)	7	0 (0.0)	7	0 (0.0)
Multiple myeloma	21	2 (9.5)	40	8 (20.0)	10	1 (10.0)	6	0 (0.0)

**Table 2:** Risk of death in unvaccinated vs. vaccinated patients in 2020–2021 in Friuli Venezia Giulia and in the province of Reggio Emilia, Italy.

	Friuli Venezia			Reggio Emilia			Overall		
	Giulia								
	HR	95% CI		HR	95% CI		HR	95% CI	
All	2.7	2.2	3.4	8.2	5.1	13.2	3.4	2.9	4.1
Sex									
Male	2.7	2.1	3.6	12.1	6	24.5	3.4	2.8	4.3
Female	2.7	1.9	3.8	3.1	1.7	5.4	2.8	2.0	3.9
Age at infection (years)									
<40	-	-	-	-	-	-	-	-	-
40–59	1.8	0.52	6	4.2	0.46	37.9	2.1	0.8	6.3
60–69	2.4	1	5.6	5.9	1	33.4	2.8	1.4	6.0
70–79	3.7	2.3	5.9	19.3	5.7	65.7	4.6	3.2	6.8
≥80	2.5	2	3.3	6.5	3.6	11.6	3.1	2.6	3.9
Time since cancer diagnosis									
<1 year	2.7	1.5	4.7	20.7	4	10.7	8.5	7.3	10.5
1–2 years	4.5	1.8	11.3	Infinite	-	-	5.3	2.6	12.1
2–5 years	2.4	1.5	3.8	5	1.9	13.6	2.8	1.9	4.2
>5 years	2.7	2.1	3.6	6.6	3.6	12.1	3.3	2.7	4.2
Tumor site									
Solid tumors	2.6	2.2	3.5	9.1	5.4	15.2	3.4	3.0	4.3
Breast	3	1.6	5.3	7.5	2.1	26.6	3.6	2.2	5.9
Prostate	2.8	1.7	4.6	8.2	2	34.1	3.2	2.1	5.0
Colorectal	2.3	1.4	3.7	18.2	3.5	95.5	2.7	1.8	4.1
Skin Melanoma	1.4	0.54	3.8	1.5	0.17	13.5	1.4	0.6	3.8
Lung and larynx	5.1	1.8	14.1	Infinite	-	-	5.8	2.5	14.8
Thyiroid	6.5	0.8	51	-	-	-	7.0	1.3	51.5
Kidney	2.2	0.6	7.5	0.46	0.04	5.6	1.2	0.1	6.5
Bladder	1.2	0.43	3.3	20.6	1.5	275	1.4	0.63	3.5
Endometrium	6.1	0.77	48.6	Infinite	-	-	10.0	4.67	52.5
Other solid cancers	3	1.7	5.2	12.4	3.2	48.8	3.7	2.4	5.9
Haematologic Malignancies	2.4	1.3	4.4	-	-	-	2.2	1.1	4.2

**Table 3:** One-year and five-year net survival (NS, %) of patients (men and women, all ages) with most frequent cancer types diagnosed in 2015–2017 (follow-up at 2021), in Friuli Venezia Giulia and in the province of Reggio Emilia, Italy.

	Friuli Venezia		Reggio Emilia	
	Giulia			
	1-Year NS	5-Year NS	1-Year NS	5-Year NS
Tumor site				
Breast	97	90	99	92
Prostate	98	94	98	88
Colorectal	82	64	84	64
Skin Melanoma	97	91	99	91
Lung	44	17	41	16
Thyroid	96	94	94	92
Kidney	83	72	84	65
Bladder	90	77	90	76
Corpus Uteri	93	79	91	78
Non-Hodgkin lymphomas	82	70	88	72
Leukemias	66	42	76	57
All sites, but skin non-melanoma	77	62	78	61

## Data Availability

The data presented in this study are available on request from the corresponding author. The data are not publicly available due to ethical and privacy issues; requests for data must be approved by the Ethics Committee after the presentation of a study protocol.

## Supplementary Materials

The following supporting information can be downloaded at: https://www.mdpi.com/article/10.3390/jpm13091333/s1, Figure S1. Distribution of death by infection’ time, by vaccination status: 79 non vaccinated and 24 vaccinated. Data from Reggio Emilia, 2021. Table S1. Distribution of causes of death between vaccinated and unvaccinated. Data from Reggio Emilia.
