# Supplementary material for: SARS-CoV-2 Infection, Vaccination and Risk of Death in People with An Oncological Disease in Northeast Italy
Source: J Pers Med. 2023 Aug 29;13(9):1333. doi: 10.3390/jpm13091333 (PMC10532764; doi:10.3390/jpm13091333)
Supplement: Supplementary file 1 [file jpm-13-01333-s001.zip › jpm-2573040-supplementary.pdf]

w	tumore	covid	altro	Total
Not vaccinated	16	45	18	79
	20.25	56.96	22.78	100.00
Vaccinated	4	12	8	24
	16.67	50.00	33.33	100.00
Total	20	57	26	103
	19.42	55.34	25.24	100.00

Table S1. Distribution of causes of death between vaccinated and unvaccinated. Data from Reggio Emilia.

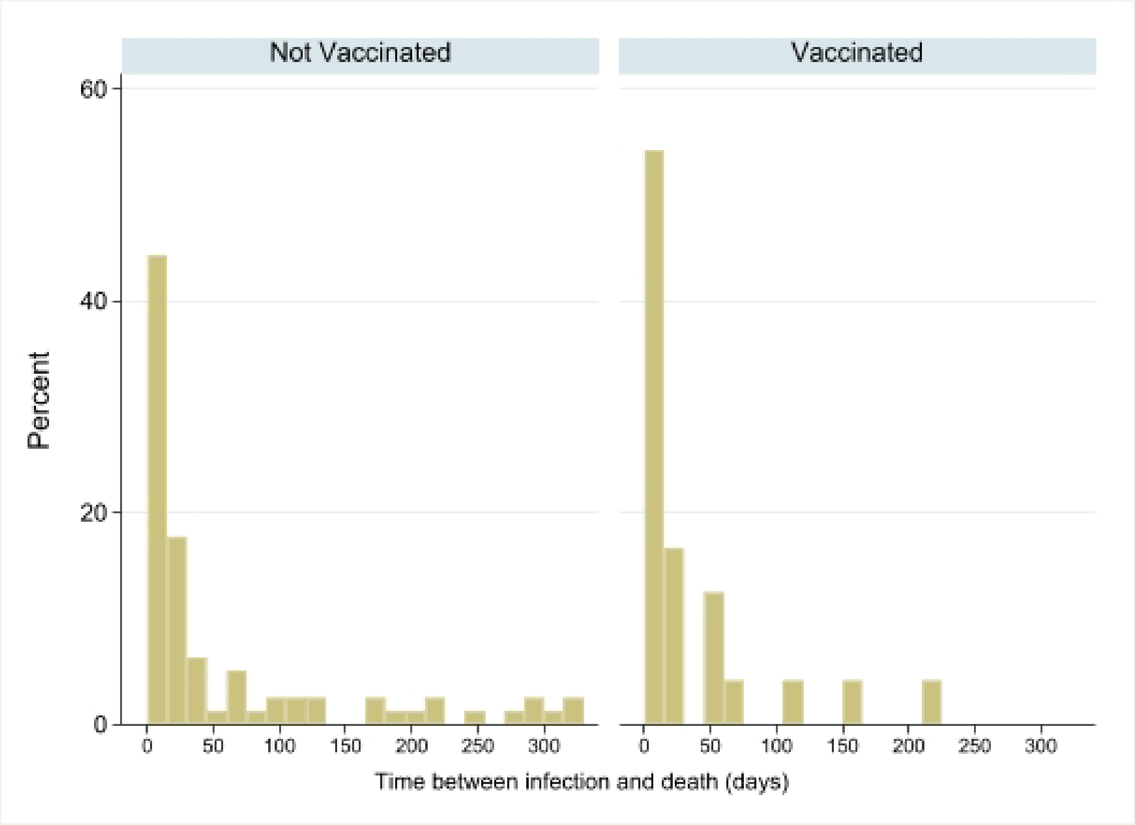

Figure S1. Distribution of death by infection' time, by vaccination status: 79 non vaccinated and 24 vaccinated. Data from Reggio Emilia, 2021.
